# Exosome-mediated renal protection: Halting the progression of fibrosis

**DOI:** 10.1016/j.gendis.2023.101117

**Published:** 2023-09-19

**Authors:** Chuanqi Liu, Qingfeng Li, Jian-Xing Ma, Baisong Lu, Tracy Criswell, Yuanyuan Zhang

**Affiliations:** a Department of Plastic and Reconstructive Surgery, Shanghai Ninth People's Hospital, Shanghai Jiao Tong University School of Medicine, Shanghai 200011, China; b Department of Biochemistry, Wake Forest School of Medicine, Winston-Salem, NC 27101, United States; c Institute for Regenerative Medicine, Wake Forest School of Medicine, Winston-Salem, NC 27157, United States

**Keywords:** Chronic kidney disease, Exosomes, Growth factors, Renal fibrosis, Stem cells

## Abstract

Renal fibrosis is a complex and multifactorial process that involves inflammation, cell proliferation, collagen, and fibronectin deposition in the kidney, ultimately leading to chronic kidney disease and even end-stage renal disease. The main goal of treatment is to slow down or halt the progression of fibrosis and to improve or preserve kidney function. Despite significant progress made in understanding the underlying mechanisms of renal fibrosis, current therapies have limited renal protection as the disease progresses. Exosomes derived from stem cells are a newer area of research for the treatment of renal fibrosis. Exosomes as nano-sized extracellular vesicles carry proteins, lipids, and nucleic acids, which can be taken up by local or distant cells, serving as mediators of intercellular communication and as drug delivery vehicles. Exosomes deliver molecules that reduce inflammation, renal fibrosis and extracellular matrix protein production, and promote tissue regeneration in animal models of kidney disease. Additionally, they have several advantages over stem cells, such as being non-immunogenic, having low risk of tumor formation, and being easier to produce and store. This review describes the use of natural and engineered exosomes containing therapeutic agents capable of mediating anti-inflammatory and anti-fibrotic processes during both acute kidney injury and chronic kidney disease. Exosome-based therapies will be compared with stem cell-based treatments for tissue regeneration, with a focus on renal protection. Finally, future directions and strategies for improving the therapeutic efficacy of exosomes are discussed.

## Introduction

Chronic kidney disease (CKD) is defined as persistent inflammation that results in progressive and irreversible damage to the renal structure and function over time.^1^ Over the past few decades, CKD has become a substantial global burden associated with increased morbidity and mortality. Currently, the prevalence of CKD is approximately 11% in high-income countries and 10%–16% in low-to middle-income countries.^2^

**Figure 1:**
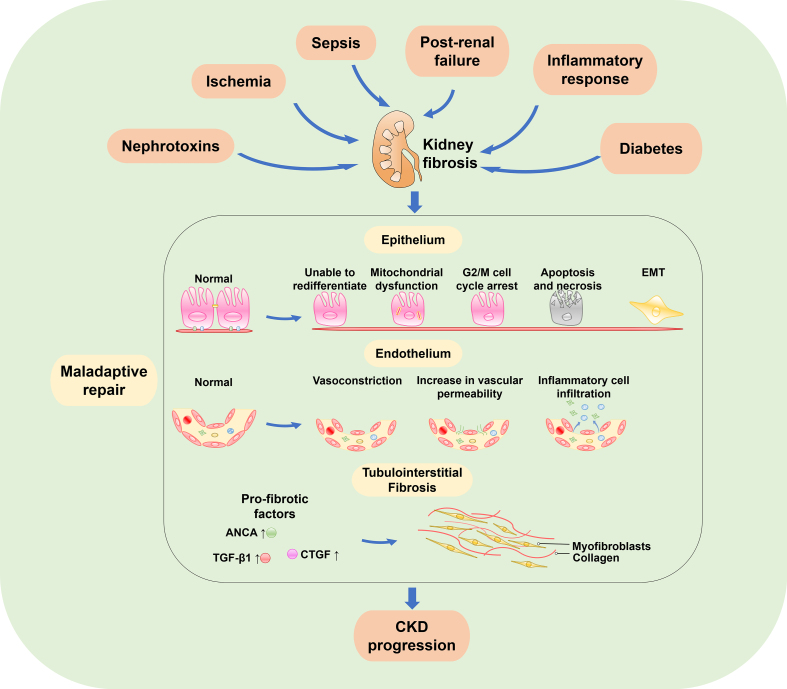
The progression of chronic kidney disease (CKD). Initiating damage to the kidney can result from complications from nephrotoxins, ischemia, sepsis, post-renal failure, inflammatory response, and diabetes leading to kidney injury. When the compensatory response is overturned by the injury, tissue repair is maladaptive. The epithelial cells become unable to redifferentiate, and undergo mitochondrial dysfunction, G2/M cell cycle arrest, apoptosis, necrosis, and EMT. Simultaneously, the endothelium experiences vasoconstriction, increased vascular permeability, and inflammatory cell infiltration. The upregulation of pro-fibrotic factors including transforming growth factor β1 (TGF-β1), connective tissue growth factor (CTGF), and anti-neutrophil cytoplasmic antibody (ANCA) leads to trans-differentiation of resident cells to myofibroblasts, which causes tubulointerstitial fibrosis. All of these changes contribute to the progression of CKD.

According to the Kidney Disease Improving Global Outcomes, the diagnosis of CKD is based on the excretory function of the kidney, measured as the glomerular filtration rate (GFR), and the extent of albuminuria, a marker of kidney barrier dysfunction.^3^^,^^4^ Early diagnosis of CKD is necessary to mitigate its progression to end-stage kidney disease and to optimize renal outcomes. The pathophysiology of CKD includes nephron loss, hypertrophy, impaired glomerular filtration, and renal fibrosis, which is characterized by glomerulosclerosis, tubular atrophy, and interstitial fibrosis (Fig. 1).^4^ Importantly, renal fibrosis represents the final pathological manifestation of CKD and is significantly correlated with CKD progression.^5^^,^^6^ Thus, it is the final target in CKD treatment.^5^ The current understanding is that renal fibrosis after injury is driven by a complex interplay between the parenchyma and various non-parenchymal cell types, including tubular epithelial cells, local myofibroblasts and immune cells, which is referred to as the fibrotic niche.^7^ Specifically speaking, transforming growth factor β (TGF-β), Notch, Wnt, and Hedgehog signaling pathways contribute to myofibroblast activation; integrin/adhesive plaque kinase/mitogen-activated protein kinase signaling promotes extracellular matrix (ECM) production and deposition; NF-κB, JAK-STAT, and TLR signaling pathways are involved in inflammatory cell activation and proinflammatory cytokine generation.^8^

The management of CKD involves nephron injury control, single-nephron hyperfiltration normalization, complication management, and kidney transplant preparation.^4^ Given the huge obstacles in kidney transplantation, current efforts are focused on minimizing nephron loss, protecting the remaining nephrons, and enhancing nephron regeneration. The most promising areas include enhancing podocyte and tubular regeneration, and blocking fibrosis and maladaptive repair.^4^

Regenerative medicine offers novel approaches for the restoration of kidney structure and function.^9^ Commonly used animal models of CKD include diabetic nephropathy (DN),^10^ unilateral ureter obstruction (UUO),^11^ ischemia-reperfusion injury (IRI),^12^ subtotal nephrectomy,^13^ and hypertensive nephropathy models.^14^ Evaluations of these therapies include histological (e.g., glomerulosclerosis and tubular interstitial fibrosis) and functional (e.g., GFR, urinary protein, plasma creatinine, plasma urea, and blood pressure) outcomes.

Mesenchymal stem/stromal cells (MSC) are the most widely studied stem cells used for renal regeneration. The safety and efficacy of MSC for ameliorating kidney injury and dysfunction have been demonstrated in hundreds of clinical trials,^15^ positioning MSC as promising candidates for CKD treatment. Nevertheless, safety concerns associated with stem cell-based therapies, including possible tumorigenesis, potential genetic instability, uncontrolled cell differentiation, and immune rejection, still exist.^16^

Stem cell culture conditioned medium has traditionally been used as a source of cytokines and growth factors that can promote lineage-specific differentiation and tissue repair.^17^ Extracellular vesicles (EVs), especially exosomes derived from cell culture medium, performed better than cell culture medium in tissue regeneration due to their high concentration of loaded cargos.^18^ Additionally, since exosomes are extracellular, they are free from safety issues associated with stem cell-based therapy.^19^ Therefore, exosomes are being investigated as a novel cell-free therapy for tissue repair and regeneration, including renal and cardiac repair^20^^,^^21^ and skin, cartilage, bone, liver, and neural regeneration.^22^, ^23^, ^24^, ^25^, ^26^ Recently, pre-clinical and clinical trials have demonstrated the efficacy of MSC-derived exosomes in renal repair and regeneration.^27^

Despite the increased interest in exosome-based cell-free therapy, challenges such as pharmacodynamic testing, targeting, fate after injection, and effect duration, still hinders its widespread adoption in clinical use. To overcome these challenges, engineered exosomes (eExos) which deliver desired cargo to improve therapeutic efficacy and prolong exosome survival in circulation are being developed. These efforts have made eExos a promising therapeutic tool for tissue-regenerative applications. This review discusses exosome-based delivery systems for tissue regeneration and compares the therapeutic outcomes of exosomes and stem cell therapy in renal protection. In addition, future strategies to improve the therapeutic efficacy of exosomes as an anti-fibrotic therapy for CKD are discussed.

## Characteristics of exosomes

### Exosome characterization and quantification

EVs are nano-to micron-sized membrane vesicles secreted by almost all cell types under physiological and pathological conditions.^28^ Currently, EVs are divided into three main categories: microvesicles (MVs), apoptotic bodies, and exosomes.^29^ Specifically, MVs are formed by direct outward budding of the cell membrane, ranging from 100 nm to 1 μm. Apoptotic bodies are released from dying cells and range in diameter from 50 nm to 5000 nm. Exosomes are formed by inward budding from the endosomal membrane and have variable diameters of approximately 40–160 nm.^28^ In general, MVs and exosomes are difficult to distinguish experimentally due to their similar biosynthetic components and overlapping sizes. Apoptotic bodies were once thought of as garbage bags. However, they have been found to deliver useful substances to healthy recipient cells, contribute to the prothrombotic state, and are used as drug delivery platforms and therapeutics.^30^

Although exosomes are defined based on their endosomal origin, tracking the EV origin once EVs are released into the extracellular compartment is still difficult. In 2018, the “Minimal Information for Studies of Extracellular Vesicles” recommended that exosomes be characterized based on their origin, size, density, and biochemical composition.^31^ Thus, the characterization of exosomes has largely relied on the methods used for exosome isolation, which have been recently optimized to improve the accuracy of exosome characterization. These techniques can be broadly classified based on their key mechanism.^32^ Examples include ultracentrifugation,^33^ sucrose or iodixanol density gradient centrifugation,^34^^,^^35^ ultrafiltration,^36^ size-exclusion chromatography,^37^ new size-based separation techniques (e.g., exosome total isolation chip, asymmetric-flow field-flow fractionation, and acoustic nanofilters),^33^^,^^38^^,^^39^ immuno-isolation technology (e.g., exosome-specific dual-patterned immuno-filtration devices and immunoaffinity),^40^^,^^41^ and exosome precipitation solutions.^42^

The rapid progress in exosome characterization technologies has resulted in a more comprehensive view of the morphological and physicochemical properties of exosomes. To obtain accurate measurements of exosome size and cargo distribution, scanning electron microscopy (SEM), transmission electron microscopy, cryo-electron microscopy, atomic force microscopy, and super-resolution microscopy are used.^43^ To detect the presence and abundance of exosomal cargos (proteins, lipids, nucleic acids, and other biomolecules), single-particle interferometric reflectance imaging combined with conventional fluorescence microscopy has been applied.^44^ However, the concentration of exosomal cargos does not perfectly correlate with the actual number of exosomes.^31^ More recently, a high-sensitivity flow cytometry instrument has been developed for the detection of exosome size and protein profiling.^45^ In the future, more neoteric technologies need to be developed to improve the accuracy of exosome characterization.

The total amount of exosomal proteins can be determined using various standard colorimetric assays (e.g., micro-bicinchoninic acid or Bradford), fluorometric assays, or global protein stain on SDS-PAGE.^31^ Total RNA can be assessed using global RNA assays such as capillary electrophoresis instruments.^46^ Specific molecule quantification can be used to quantify exosomes through enzyme-linked immunosorbent assay^47^ or a colorimetric aptasensor based on carbon nanotubes.^48^ Providing a deeper insight into the physical characteristics of exosomes, these techniques are valuable additions to the above-mentioned quantification methods.

The dose of exosomes used varies according to the intended application (Table 1). Therefore, several techniques have been used to quantify exosomes (Table 2).^49^ Light scattering technologies, such as dynamic light scattering, SEM, and nanoparticle tracking analysis, can be used for counting particles.^50^ Larger (>100 nm) and smaller (<100 nm) exosomes can be quantified based on standard flow cytometry^51^ and nano-flow cytometry,^52^ respectively. Resistive pulse sensing can be used to quantify a wide range sizes of exosomes.^53^ Additionally, atomic force microscopy and field emission SEM (FESEM) have also been used to examine the nanoscale structures of exosomes under varying forces.^54^

**Table 1:** Applications of exosomes or eExos as therapeutic agents for kidney disease.

Exosome Source	Animal model	Cargo	Signaling pathway	Administration (dose)	Latest time of detection	Main findings
**BMSC**						
Xenogeneic (human)	Rat, Nephrectomy + IRI, AKI	n/a	n/i	Intravenous (Single: 30 μg)	Detectable at 2 h, 6 h, but not at 24 h	Enhanced tubular cell proliferation, and inhibited of apoptosis, and reduced fibrosis^125^
Allogeneic	Mouse, UUO, AKI	miR-29, miR-30, miR-210-3p	TGF-β	Intravenous (Single: 30 mg)	n/i	Protected against EMT and renal failure^128^
Xenogeneic (human)	Mouse, cisplatin, AKI	n/a	MAPK, Akt	Intravenous (Single: 150 μg/100 g)	Detectable at 12d	Improved kidney function and reduced inflammation^126^
Allogeneic	Mouse, STZ-DN, CKD	n/a	n/i	Subcapsular injection (Single: 5.3 × 10^7^)	Detectable at 96 h	Renoprotective effects with anti-apoptotic, anti-inflammatory potential^92^
Allogeneic	Mouse, UUO, CKD	miR-34c-5p	TGF-β-SMAD2/3, PDGF-ERK1/2, EGFR-ERBB3, BMP7-SMAD6/7	Intravenous (Single: 100 μg)	Peaked at 48 h, and became undetectable at 10 days	Inhibited renal interstitial fibrosis^129^
Xenogeneic (human)	Mouse, hypertensive nephropathy, CKD	n/a	n/i	Intravenous (Multiple: 25 μg/d for 7 days)	n/i	Reduced tissue inflammation and fibrosis^130^
Xenogeneic (human)	Mouse, STZ-DN, CKD	miRNAs	TGF-β, IGF-1, EGFR, PDGFR	Intravenous (Multiple: 1 × 10^10^/w for 4 weeks)	n/i	Inhibited fibrosis and prevented CKD progression^131^
**UMSC**						
Xenogeneic (human)	Rat, Cisplatin, AKI	n/a	p38/MAPK, Erk1/2	Renal capsule injection (Single: 200 μg)	Detectable at 24 h	Promoted cell proliferation, and reduced oxidative stress and cell apoptosis^132^
Xenogeneic (human)	Rat, IRI, AKI	miR-30b/c/d	n/i	Intravenous (Single: 100 μg)	Detectable at 3 h	Reduced mitochondrial damage^133^
Xenogeneic (human)	Mouse, IRI, AKI	miR125b-5p	p53	Intravenous (Multiple: 50 μg or 100 μg at 0 h and 24 h)	n/i	Ameliorated ischemic AKI and promoted tubular repair^119^
Allogeneic	Mouse, STZ-DN, CKD	n/a	TGF-β1/Smad2/3, PI3K/Akt, MAPK	Intravenous (Single: dose n/s)	n/i	Inhibited renal fibrosis in DN^134^
Xenogeneic (human)	Rat, UUO, CKD	n/a	p38MAPK/ERK	Injection via renal artery (Single: 200 μg)	n/i	Protected against ROS^135^
Xenogeneic (human)	Rat, UUO, CKD	CK1δ, β-TRCP	Hippo-YAP	Intravenous (Multiple: 10 mg/kg on day 6, 9, 12)	Detectable at 24 h	Attenuated renal fibrosis^136^
**ASC**						
Xenogeneic (human)	Mouse, Sepsis, AKI	n/a	SIRT1	Intravenous (Single: 100 μg)	n/i	Preserved renal function, and decreased inflammation^138^
Allogeneic	Rat, IRI, AKI	n/a	n/i	Intravenous (Single: 100 μg)	n/i	Alleviated kidney impairment from IRI^139^
Autologous	Porcine, MetS + RAS, CKD	IL10	n/i	Injection via renal artery (Single: dose n/s)	Peaked at 2d, and remained at 2% by 4w	Attenuated renal inflammation, fibrosis, and oxidative stress^14^
Autologous	Swine, MetS + RVD, CKD	Angiogenic proteins	n/i	Injection via renal artery (Single: dose n/s)	Detectable at 4w	Attenuated renal apoptosis, and fibrosis^137^
Autologous	Mouse, DN, CKD	miR-486	Smad1/mTOR	Intravenous (Multiple: dose n/s)	n/i	Ameliorated DN symptom markedly^196^
**iPSC-MSC**						
Xenogeneic (human)	Rat, IRI, AKI	SP1	SP1–SK1–S1P	Intravenous (Single: 1 × 10^12^)	n/i	Reduced necroptosis and supported tissue recovery^144^
Xenogeneic (human)	Mouse, UUO, CKD	n/a	Sirtuin 6/β-catenin	Intravenous (Single: 1 × 10^11^)	n/i	Reduced inflammation and fibrosis, and improved kidney function^11^
**USC**						
Xenogeneic (human)	Rat, IRI, AKI	miR-146a-5p	NF-κB	Intravenous (Single: 20 μg)	n/i	Protected against IRI-induced renal damage^147^
Xenogeneic (human)	Mouse, STZ-DN, CKD	BMP-7, VEGF, TGF-β, angiogenin	n/i	Intravenous (Multiple: 100 μg/w for 12 weeks)	n/i	Inhibited of podocyte apoptosis and promoted of vascular regeneration and cell survival^10^
Xenogeneic (human)	Rat, DN, CKD	miR-16-5p	n/i	Intravenous (Multiple: 100 μg/w for 12 weeks)	n/i	Protected against podocytes apoptosis^148^
**Other sources**						
**HLSC**						
Xenogeneic (human)	Mouse, AA-induced, CKD	n/a	PDGF, FGF, TGF-β	Intravenous (Multiple: 1 × 10^10^/w for 4 weeks)	n/i	Reduced interstitial fibrosis and tubular necrosis^197^
**Renal tubular cells**						
Allogeneic	Rat, IRI, AKI	n/a	HIF	Intravenous (Multiple: 100 μg at 24 h and 48 h)	n/i	Limited fibrotic gene activation and decreased renal fibrosis^198^
**Exos**						
***MiR-let7c-MSC***						
Xenogeneic (human)	Mouse, UUO, CKD	miR-let7c	TGF-β	Intravenous (dose and frequency n/s)	n/i	Decreased renal fibrosis^149^
***Anti-let-7i-5p-MSC***						
Xenogeneic (human)	Mouse, UUO, CKD	Anti-let-7i-5p	TSC1/mTOR	Intravenous (Multiple: 50 μg twice weekly for 4 weeks)	Detectable for 2 days	Anti-fibrosis^151^
***MiR-374a-5p-BMSC***						
Species n/s	Mouse, UUO, CKD	miR-374a-5p	MAPK6/MK5/YAP	Intravenous (Multiple: dose n/s, twice weekly for 4 weeks)	n/i	Prevented progression of renal fibrosis^150^
***GDNF-ASC***						
Xenogeneic (human)	Mouse, UUO, CKD	GDNF	SIRT1/eNOS	Intravenous (Single: 200 μg)	n/i	Ameliorated renal fibrosis^152^
***MiR-29-muscle satellite cells***						
Allogenic	Mouse, UUO, CKD	miR-29	Yin Yang 1, TGF-β	Intravenous (Multiple: dose n/s, once a week for 2 weeks)	Detectable at 7, 14, and 21 days	Decreased renal fibrosis^153^

**Abbreviations**: ASC, adipose stem cells; BMSC, bone marrow mesenchymal stem/stromal cells; IRI, ischemia-reperfusion injury; AKI, acute kidney injury; UUO, unilateral ureteral obstruction; CKD, chronic kidney disease; EMT, epithelial to mesenchymal transition; ROS, reactive oxygen species; TGF-β, transforming growth factor beta; STZ, streptozotocin; SP1, specificity protein 1; n/a, not applicable; n/i, not investigated; n/s, not specified, USC, urine-derived stem cells.

**Table 2:** Techniques for quantifying exosomes.

Category	Techniques	Characteristics
Particles	Scanning electron microscopy and nanoparticle tracking analysis	Analyzing size and integrity of exosomes^50^
	Dynamic light scattering	Cannot Safely determined exosome size^50^
	Standard flow cytometry	Quantifying large exosomes^199^
	High resolution or image-based flow cytometry	Quantifying small exosomes^200^
	Resistive pulse sensing	Quantifying a wide range sizes of exosomes^53^
	FESEM	Interpreting the nanoscale structures^54^
Protein	BCA, Bradford, fluorometric assays, global protein stain	Potential overestimation of exosome quantification^31^
RNA	Global RNA assays	Not recommended for quantification or purity assessment^31^
Specific molecule	ELISA, colorimetric aptasensor based on carbon nanotubes, etc.	Data on cargo in exosomes^47^^,^^48^

**Abbreviations**: FESEM, field emission scanning electron microscopy; BCA, bicinchoninic acid; ELISA, enzyme-linked immunosorbent assay; RNA, ribonucleic acid.

Characterization and measurement of exosomes have provided a better understanding of their physical properties and composition. Exosomes tend to be unilamellar spherical EVs (approximately 40–160 nm), with a structure similar to that of cells.^55^^,^^56^ However, exosomes with larger diameters (>160 nm) and unusual membrane structures, such as multiple membranes or tubule-like morphologies, have also been observed.^55^ These unusual morphologies are generated by physical force^57^ or contaminating elements in the exosome preparation processes.^58^ Exosome density is 1.1–1.2 g/ml, which is affected by the protein-lipid ratio.^59^ In general, exosome size, shape, and density vary over a wide range due to their large cargo composition variation; these measurements cannot be used to define exosome features.

### Exosome biogenesis, secretion, uptake, and intercellular communication

**Figure 2:**
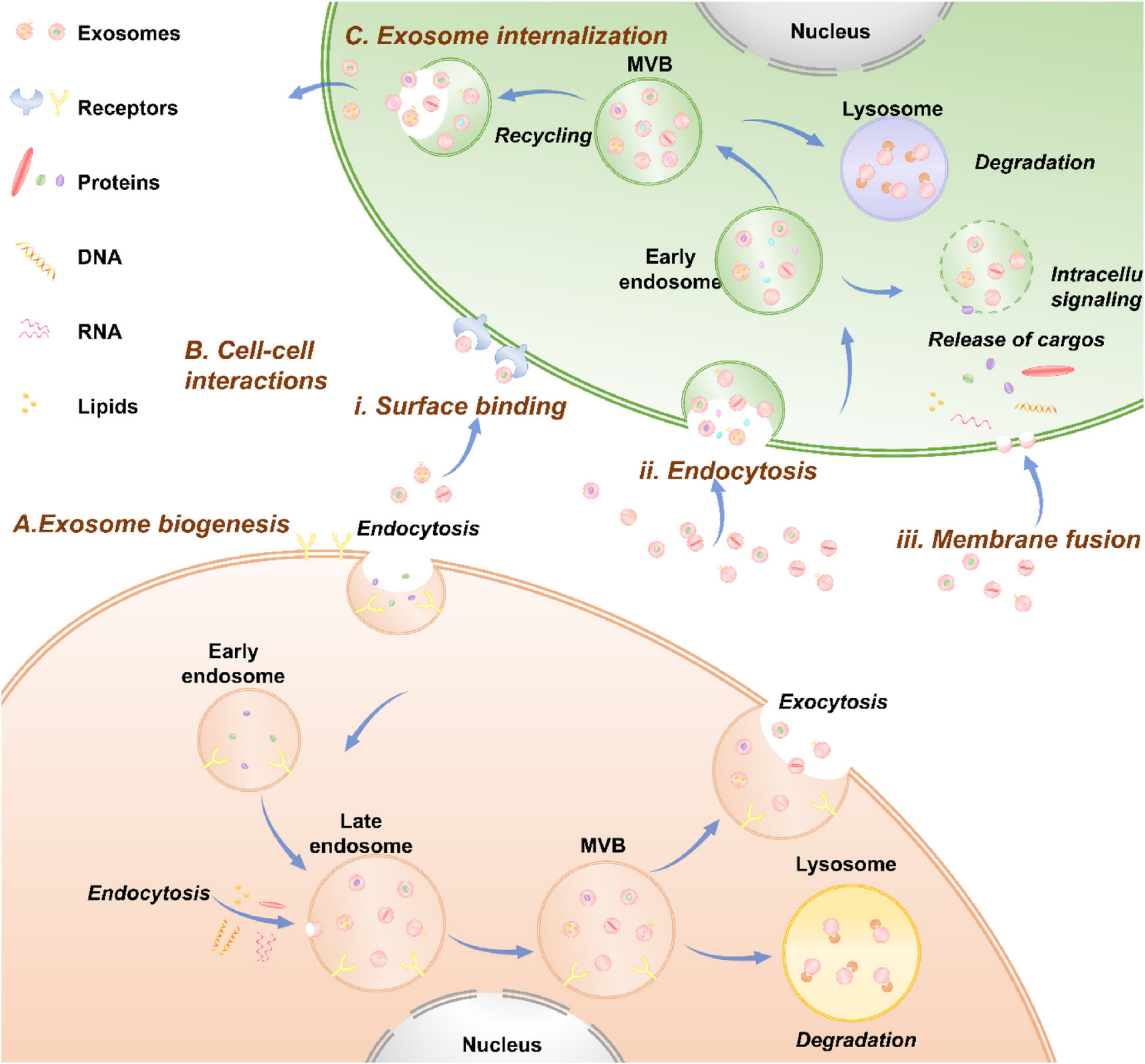
Biogenesis of exosomes and intercellular communication. **(A)** Exosome biogenesis and secretion. The invagination of the plasma membrane forms the early endosome membrane. The second invagination of the endosomal membrane generates the intraluminal vesicles (ILVs). The early endosomes gradually mature into multivesicular bodies (MVBs) engulfed with several ILVs (future exosomes). MVBs can subsequently fuse with the plasma membrane, releasing exosomes to the extracellular space; or fuse with lysosomes, undergoing degradation as a part of catabolic processes. **(B)** Cell–cell interactions mediated by exosomes. The processes are achieved through surface binding, membrane fusion, and endocytosis. **(C)** Exosomes internalized by recipient cells. Exosomes may release their content into the cytoplasm, be re-secreted or targeted to the lysosome for degradation.

Three classical steps of exosome biogenesis in donor cells are described here (Fig. 2A)^60^: 1) Establishment of the endosomal system: A cup-shaped structure is formed through plasma membrane invagination, resulting in early sorting endosome formation^28^; 2) Formation of intraluminal vesicles (ILVs): the second invagination of the endosomal membrane generates ILVs. Early sorting endosomes mature into late sorting endosomes, eventually giving rise to multivesicular bodies (MVBs) engulfing several ILVs (future exosomes)^28^; and 3) Release of the ILVs/exosomes into the extracellular space: MVBs fuse with the plasma membrane, subsequently releasing ILVs to the extracellular environment.^28^ MVBs may also fuse with lysosomes, where they undergo degradation due to hydrolase exposure as a part of catabolic processes.

Exosome secretion and uptake between donor and recipient cells are complex. Although the mechanisms of exosome uptake are still incompletely characterized, uptake processes involve^61^: targeting recipient cells, entering recipient cells, and delivering cargo to recipient cells. Exosome secretion is regulated by Rab GTPases, soluble N-ethylmaleimide-sensitive factor attachment protein receptor proteins, and cytoskeletal elements.^61^ Although exosomes can deliver transmembrane signals at the surfaces of recipient cells, their main function is to transmit molecules to recipient cells.^61^ The entry of exosomes into recipient cells, prior to cargo delivery, occurs via endocytosis, membrane fusion and surface binding (Fig. 2B).^62^^,^^63^ Exosomes have three possible fates once they enter the recipient cells (Fig. 2C): 1) the majority of ILVs fuse with the endosome membrane, release their cargos into the cytoplasm, and perform their functions; 2) some fuse with lysosomes and are degraded; and 3) some are released into the extracellular environment via exocytosis, which may explain why exosomes are quickly cleared in damaged tissues.^28^

Exosomes can mediate intercellular and interorgan communication through local paracrine or distal systemic effects.^64^ Once exosomes are secreted into the extracellular space, they gain access to the circulatory system and can be delivered to distal cellular sites where they can undergo endocytosis or fuse into targeted cells. This phenomenon has been observed in adipocytes, endothelial cells, and pancreatic beta cells.^64^ Endogenous exosomes play several roles *in vivo*. For example, endogenous dendritic cell exosomes have been showed to exacerbate the inflammatory response in a mouse model.^65^ Exosomes produced by endogenous immune cells play an immunomodulatory role after acute myocardial infarction.^66^ Furthermore, in some complex heterogeneous disorders, such as obesity-induced chronic inflammation, exosomes are involved in complicated multiorgan communications.^67^ Importantly, secreted exosomes and paracrine signaling play pivotal roles, especially in renal dysfunction such as DN.^68^

### Exosome composition

Cargo sorting into ILVs is mediated by endosomal sorting complex required for transport (ESCRT)-dependent^69^ and -independent^70^ mechanisms. According to data from several high-throughput studies, exosomes are highly heterogeneous in composition (proteins, nucleic acids, and lipids).^71^ Additionally, exosome composition largely depends on cellular origin and the physio-pathological state.

#### Exosomal proteins

Exosomes contain a variety of proteins that are involved in several cellular processes including cell adhesion, structural dynamics, membrane fusion, signal transduction, and metabolism.^72^ They contain both representative proteins of parental cells and common proteins irrespective of cell types. These exosome proteins are divided into four categories: transmembrane, lipid-anchored, peripherally associated, and soluble proteins.^55^

1) Transmembrane proteins act as cell surface signaling molecules, functional receptors, and signaling molecules.^55^ Tetraspanins are transmembrane proteins that span the plasma membrane four times and promote trafficking, stability, function, and oligomerization of other membrane proteins.^73^ Escola et al^74^ established that four tetraspanins (CD63, CD81, CD82, and CD37) are highly enriched in exosomes. CD81 and CD63, along with other tetraspanins, such as CD9, have become the most common markers of exosomal proteins.^73^
2) Lipid-anchored membrane proteins can be anchored to the outer^75^ (CD39, CD73, CD55, CD59, and glypican-1) or inner^76^ (prenylated small GTPases [e.g., Rabs], myristoylated signaling kinases [e.g., Src], palmitoylated membrane proteins, and Gag proteins [e.g., activity-regulated cytoskeletal protein]) membrane.
3) Peripherally associated membrane proteins, such as wingless (Wnt) proteins,^77^ bone morphogenetic proteins (BMPs),^78^ TGF-β,^79^ and certain scaffolding proteins (e.g., ezrin-radixin-moesin proteins),^80^ are involved in cell signaling.
4) Soluble proteins in the exosome lumen include a large portion of cellular abundant proteins (e.g., tubulin) and a small portion of heterologous proteins involved in exosome secretion.^81^

Exosomal proteins can also be specific to the cell type of origin. For example, CD37 and CD53 can be used to identify exosomes from leukocytes; platelet-endothelial cell adhesion molecule-1 is specific to endothelial cell exosomes; and CD90 is a representative marker for MSC-derived exosomes.^31^ Considering the complexity of exosomal proteins, the International Society of Extracellular Vesicles recommends that the characterization of exosomes should include at least three protein markers: one transmembrane (e.g., CD81, CD82, CD37, and CD63), cytosolic (e.g., syntenin, TSG101, and ALIX), and negative marker (e.g., albumin) protein.^31^

#### Exosomal nucleic acids

Exosomes contain both RNA and DNA nucleotides. These nucleic acids can be delivered from the donor cells to recipient cells or tissues. RNAs include both coding mRNAs and non-coding RNAs, such as microRNAs (miRNAs), ribosomal, small nuclear, and transfer RNAs.^55^ These RNAs are actively sorted into exosomes via different mechanisms. MiRNAs are believed to be the major components mediating regulatory activity. Exosomal miRNAs play a crucial role in exosome-mediated cell communication during cellular proliferation, inflammatory response, and glucose metabolism regulation.^82^

Some exosomal RNA subsets are cell- or tissue-specific, while others are present in exosomes regardless of their cellular origin. It is hypothesized that the delivered exosomal RNA cargo targets specific mRNA and results in functional modulation.^83^ Exosomal DNA can be single-stranded,^71^ double-stranded,^84^ genomic,^85^ or mitochondrial DNA.^86^ Exosomal DNA may play a role in cellular DNA quality control and serve as biomarkers of numerous diseases and chemotherapeutic resistance.^55^

#### Exosomal lipids

Exosomes are enriched in lipids, including sphingolipids, cholesterol, glycosphingolipids, phosphatidylserine, saturated fatty acids, and ceramide.^87^ These lipids are critical structural components of exosome membranes. Additionally, exosomal lipids play an important role in protecting exosome morphology, as well as in exosome biogenesis, secretion, and homeostasis regulation in recipient cells.^88^ Lipids such as ceramide play an important role in driving the budding of ILVs and exosome secretion.^70^

### Contributions of exosomes to tissue repair

Implementation of exosomes as cell-free therapy for tissue repair prevents the side effects of cell therapy. Although exosomes may contribute to tissue fibrosis (e.g., exosomes from hepatic stellate cells promote liver fibrosis,^89^ exosomes containing Epstein–Barr viral product promote nasopharyngeal carcinoma fibrosis^90^), it is unclear whether exosomes are involved in this process. However, MSC-derived exosomes possess anti-apoptotic, anti-inflammatory, anti-oxidant, anti-fibrotic, anti-tumorigenic, pro-angiogenic, and pro-regenerative effects.^91^ Nagaishi et al^92^ reported that exosomes could suppress apoptosis and inflammatory cell infiltration, contributing to the improvement of DN. These functions have been implicated in tissue repair, making exosomes the prime candidates for regenerative medicine applications.

Exosomes have prominent advantages as endogenous drug-delivery systems. They have excellent biocompatibility and low immunogenicity, and can penetrate biological barriers (e.g., the blood–brain barrier) and home to target cells or tissues.^83^ Thus, they are ideal carriers for the delivery of proteins, miRNAs, small-interfering RNAs, and other biomacromolecules.

It is now widely accepted that tissue- or cell-specific cargos render exosomes a novel cell–cell communication mechanism. This concept is based on the interactions between parental exosomes and recipient cells; exosomes influence recipient cell behaviors and phenotype features.^93^ For example, cardiomyocyte progenitor cell (CPC)-derived exosomes can enhance endothelial cell migration,^94^ and the miRNAs in CPC-derived exosomes play a critical role in improving cardiac function and reducing cardiac fibrosis.^95^ Increasing evidence has demonstrated the protective effect of exosomes against renal injury or prevention of renal fibrosis.^96^

## Exosomes and stem cells as therapeutic agents

### Anti-inflammatory and anti-fibrotic therapies

Various strategies to protect the kidneys from injury have been investigated (Table 3). Anti-inflammatory strategies target angiotensin-converting enzyme (ACE),^97^ angiotensin receptor,^98^ TGF-β1,^99^ Wnt/β-catenin,^100^ interleukin (IL)-17A,^101^ and bromodomain and extra-terminal domain (BET) family proteins.^102^ On the other hand, anti-fibrotic strategies include inhibition of TGF-β1^103−106^ and connective tissue growth factor (CTGF).^107^^,^^108^ Notably, oxidative stress is associated with kidney disease progression.^109^ Evidence from preclinical and clinical studies suggests the improvement of acute kidney injury (AKI) or CKD with antioxidant agents,^110^^,^^111^ such as vitamins C and E. Curcumin upregulates antioxidant enzymes, and other cytoprotective proteins (e.g., superoxide dismutase), resulting in indirect antioxidative activity.^112^, ^113^, ^114^, ^115^, ^116^, ^117^, ^118^ Strategies for promoting renal tubule repair have been well-documented. For example, exosomal miR-125b-5p can ameliorate ischemic AKI and promote tubular repair via the miR-125b-5p/p53 pathway.^119^

**Table 3:** Therapeutics with known protective effects against kidney disease.

Target	Agent	Patients/Animal model	Main Findings
Anti- inflammation			
*ACE*	ACE inhibitor	Advanced CKD patients	Reduced plasma levels of TNF-α and C-reactive protein^97^
*Angiotensin receptor*	Losartan	Hemodialysis patients	Reduced pro-inflammatory CD14^+^CD16^+^ monocytes^98^
*TGF-β1, Wnt/β-catenin*	Klotho	UUO mice	Prevented tubulointerstitial and glomerular injury and fibrosis^99^^,^^100^
*IL-17A*	Anti-IL17A neutralizing antibody	Hypertension-induced kidney injury mice	Associated with anti-inflammatory effects in the kidney^101^
*BET*	BET inhibitor (JQ1)	UUO and AngII infusion mice	Reduced renal inflammation and ameliorated renal damage^102^
Anti-fibrosis			
*TGF-β1*	Pirfenidone	DN and FSGS patients	Decreased fibrosis and reduced the loss of eGFR^103^
	Fresolimumab	FSGS patients	Corresponded to a blunting of renal fibrogenesis in Phase I and II clinical trials^104^^,^^105^
*TGF-β1, Notch*	JMJD3	UUO and SNx mice	Suppressed TGF-β1 and Notch signaling pathways^106^
*CTGF*	Monoclonal anti-CTGF antibody	DN patients	Showed inefficiency in Phase I clinical trial^107^
	Anti-CTGF antibody FG-3019	UUO mice	Showed anti-fibrotic effects^108^
Antioxidant stress			
*TGF-β, ROS*	Vitamin C	Hyperuricemic nephropathy rats	Delayed the progression of hyperuricemic nephropathy^112^
*ROS*	Vitamin E	CKD patients	Vitamin E combined with 0.9% saline infusion performed better than placebo^114^, ^115^, ^116^
*Antioxidant enzymes and cytoprotective proteins*	Curcumin	5/6NX rats	Reversed established oxidant stress glomerular hypertension and hyperfiltration in 5/6NX rats^118^
Renal tubule repair			
*p53*	Exosomal miR-125b-5p	IRI-induced AKI rats	Ameliorated ischemic AKI and promoted tubular repair^119^

**Abbreviations**: CKD, chronic kidney disease; ACE, angiotensin converting enzyme; TNF-α, tumor necrosis factor-α; TGF-β1, transforming growth factor beta 1; UUO, unilateral ureteral obstruction; IL-17A, interleukin 17A; BET, bromodomain and extra-terminal domain; AngII, Angiotensin II; DN, diabetic nephropathy; FSGS, focal segmental glomerulosclerosis; eGFR, estimated glomerular filtration rate; JMJD3, Jumonji domain containing-3; SNx, 5/6 nephrectomy; CTGF, connective tissue growth factor; ROS, reactive oxygen species; NX, nephrectomy; IRI, ischemia-reperfusion injury; AKI, acute kidney injury.

In general, MSC-derived or eExos are the most commonly tested therapeutic agents for alleviating renal dysfunction or fibrosis.^120^ In fact, MSC-derived exosomes provide this therapeutic benefit for a wide spectrum of fibrotic states.^121^ Additionally, eExos loaded with anti-inflammatory and anti-fibrotic agents (such as pigment epithelium-derived factor) have been explored as a means to attenuate kidney,^122^ liver,^123^ and cardiac fibrosis.^124^

### Exosomes as therapeutic agents in renal fibrosis (Table 1)

#### Bone marrow MSC (BMSC)-derived exosomes

Therapeutic strategies for preventing and attenuating renal fibrosis based on BMSC-derived exosomes (BMSC-Exos) has been previously described. Gatti et al^125^ reported that BMSC-Exos protected rats from AKI by stimulating tubular cell proliferation, inhibiting apoptosis, and reducing fibrosis. Likewise, Ullah et al^126^ proved that BMSC-Exos significantly reduced inflammation and improved kidney function. Furthermore, Yin et al^127^ showed that BMSC-Exos could prevent the epithelial-mesenchymal transition (EMT) of renal tubular epithelial cells *in vitro* by inhibiting TGF-β1 signaling. Similarly, a study by He et al^128^ demonstrated that BMSC-Exos were able to inhibit EMT and the consequent renal failure. Other studies have demonstrated the anti-apoptotic and anti-inflammatory functions of BMSC-Exos in a streptozotocin (STZ)-DN mouse model,^92^ and anti-fibrotic effects in UUO,^129^ hypertensive nephropathy,^130^ and DN mouse models.^131^

#### Umbilical cord MSC (UMSC)-derived exosomes

UMSC-derived exosomes (UMSC-Exos) are also promising candidates for renal fibrosis treatment. Zhou et al^132^ reported that UMSC-Exos could repair renal injury by promoting cell proliferation and reducing oxidative stress and apoptosis in an AKI rat model. Gu et al^133^ reported that UMSC-Exos protected against IRI, and Cao et al^119^ further demonstrated that UMSC-Exos promoted tubular repair by inducing cell cycle arrest and apoptosis through the miR-30/p53 pathway. In addition, Xiang et al^121^ demonstrated that UMSC-Exos decreased the production of pro-fibrotic and pro-inflammatory factors, including TGF-β, IL-6, IL-1β, and tumor necrosis factor (TNF)-α in renal tubular and glomerular endothelial cells. These results confirmed that UMSC-Exos are capable of inhibiting renal fibrosis and inflammation, preventing CKD progression, and improving renal function *in vivo*. Moreover, several groups have identified the paracrine signaling mechanisms involved in the anti-fibrotic effects mediated by UMSC-Exos, including regulation of the reactive oxygen species (ROS)-mediated P38/mitogen-activated protein kinase (MAPK)/extracellular signal-regulated kinase (ERK) signaling pathway,^134^^,^^135^ and yes-associated protein 1 (YAP) degradation via CK1δ and β-transducin repeat-containing protein.^136^

#### Adipose-derived MSC (ASC)-derived exosomes

ASC-derived exosomes (ASC-Exos) have anti-fibrotic effects in different animal models of renal injury.^14^^,^^137^ ASC are commonly used because of their ease of isolation. Gao et al^138^ reported that ASC-Exos protected against renal injury in a sepsis-induced AKI mouse model. Furthermore, Lin et al^139^ reported that ASC-Exos combined with ASC markedly alleviated kidney impairment caused by IRI through decreased inflammation and apoptosis and increased angiogenesis. Regarding CKD, Eirin et al^14^^,^^137^ reported that ASC-Exos attenuated renal inflammation, fibrosis, and oxidative stress in a porcine CKD model. Moreover, they demonstrated that the protective effects of exosomes in the kidney could be blunted by decreased expression of IL10.^14^^,^^137^ Additionally, Jin et al^140^ illustrated that ASC-Exos ameliorated DN symptoms by inhibiting podocyte apoptosis via the Smad1/mTOR signaling pathway.

#### Induced pluripotent stem cells (iPSC)-MSC-derived exosomes

All previously described MSC were derived from tissues, showing decreased proliferation and regenerative potential after expansion.^141^ MSC can also be derived from iPSC (iPSC-MSC), which are capable of prolonged proliferation and expansion with high efficiency *in vitro*.^142^ iPSC-MSC had equivalent anti-apoptotic and anti-fibrotic effects as tissue-derived MSC in an adriamycin-induced mouse model of nephropathy.^143^ Likewise, iPSC-MSC-derived exosomes had an anti-necroptosis effect against renal IRI,^144^ reducing inflammatory responses and renal fibrosis, and improving kidney function in a UUO mouse model.^11^ Notably, this protective effect might be mediated by increased sirtuin 6 and decreased β-catenin expression.^11^ In contrast, a recent study showed that allogeneic iPSC exosomes were less effective and less viable than their autologous counterparts.^145^ Thus, a comparison of the effects of iPSC-MSC-derived exosomes and autogenic stem cell-derived exosomes is required.

#### Urine-derived stem cells (USC)-derived exosomes

Compared to other stem cells, USC are relatively free of ethical concerns, easily accessible, and consistently reproducible.^146^ Recent data have demonstrated that USC-derived exosomes (USC-Exos) had protective effects against IRI-induced renal damage via exosomal miR-146a-5p, which subsequently inhibited nuclear factor kappa B signaling pathway activation.^147^ Interestingly, Jiang et al^10^ showed that USC-Exos protected the kidney through the inhibition of podocyte apoptosis and promotion of vascular regeneration in rats. Additionally, Duan et al^148^ reported that the overexpression of miR-16-5p in USC-Exos provided podocyte protection against high glucose-induced DN in rats. Thus, USC-Exos are emerging as attractive, cell-free, and anti-fibrotic therapies.

#### eExos

eExos are designed and more frequently used to deliver specific cargo to identified targets. Administration of eExos has resulted in beneficial therapeutic effects in different renal fibrosis models. Treatment with human BMSC-Exos carrying exogenous miR-let7c,^149^ miR-374a-5p,^150^ or antagomir against let-7i-5p^151^ resulted in decreased renal fibrosis by targeting TGF-βR1 signaling or the MAPK6/MK5/YAP axis, respectively. In a UUO mouse model, Chen et al^152^ reported that ASC-Exos transfected with glial cell-derived neurotrophic factor (GDNF) ameliorated renal fibrosis by activating the SIRT1/eNOS signaling pathway. Wang et al^153^ employed eExos-encapsulated miR-29 to attenuate renal fibrosis through the inhibition of the Yin Yang 1 and TGF-β-associated signaling pathways. These data confirmed the utility of eExos in delivering therapeutic miRNA cargo to decrease fibrosis associated with CKD.

### Comparisons of therapeutic outcomes of stem cells, exosomes and eExos

Unfortunately, very few experimental studies have made direct efficacy comparisons between exosome-based therapy and its counterpart stem-cell based therapy. Of note, Zhao et al^154^ found that EVs derived from ASC better preserved the cellular integrity of the kidney than ASC alone. In another study, Ishiy et al^155^ reported that rats treated with ASC-Exos, as compared to ASC-treated group, showed a reduction in pro-fibrotic proteins including Col I and TGF-β, with a corresponding increase in anti-inflammatory cytokine IL-10. More studies should be conducted to determine the benefits and limitations of stem cell- and exosome-based therapies for different tissue repairs (Table 4).

**Table 4:** Advantages and disadvantages of stem cells, exosomes- and eExos for CKD therapy.

	Benefits	Limitations
Stem cells	1) Preserve kidney structure and renal function	1) Biodistribution or engraftment at undesired locations
	2) Can be genetically modified	2) Adventitious agent or microorganism contamination
		3) Potential immune and inflammatory responses when allogeneic stem cells are used
		4) Potential risk of tumorigenicity or oncogenesis
		5) Risks of emboli formation when a large number of cells are intravenously injected in short time period
		6) Mass production is difficult
Exosomes	1) Preserve kidney structure and renal function	Biodistribution or engraftment at undesired locations
	2) Resistant to side effects of inflammatory immune response, increasing half-life *in vivo*	
	3) Can be preserved and stored for a long period	
	4) Relatively easy to become off-the-shelf products based on mass-production and dosage evaluation	
eExos	1) Preserve kidney structure and renal function	Biodistribution or engraftment at undesired locations
	2) Resistant to side effects of inflammatory immune response, increasing half-life *in vivo*	
	3) Can be preserved and stored for a long period	
	4) Relatively easy to become off-the-shelf products based on mass-production and dosage evaluation	
	5) Can be engineered to display a wide range of targeting protein/peptide ligands or directly encapsulate agents for enhanced cargo delivery	

**Abbreviations**: eExos, engineered exosomes; CKD, chronic kidney disease.

#### Stem cell-based therapy

Stem cells are specialized cells that can self-renew and differentiate into mature tissue.^156^ Generally, they are categorized based on their differentiation potential or potency. There are five stem cell types: 1) totipotent stem cells can differentiate into all the cells of an organism plus the extraembryonic tissues (e.g., placenta); 2) pluripotent stem cells (PSC) possess the ability to differentiate into all the cells of an organism, but not the extraembryonic structures. Two examples of PSC include embryonic stem cells (ESC) which are derived from the inner cell mass of a preimplantation blastocyst, and induced PSC (iPSC) generated from genetically manipulated somatic cells. The clinical use of iPSC in cell therapy faces potential immune rejection, tumorigenicity, cell population heterogeneity, genomic instability, ectopic differentiation, and short life-term concerns^16^; 3) multipotent stem cells are lineage-restricted to a specific germ layer. Most adult stem cells tested for preclinical and clinical use are multipotent stem cells. A major benefit of MSC is that they originate from a wide range of sources, including bone marrow, adipose tissue, umbilical cord, and placenta (amniotic fluid)^120^; 4) oligopotent stem cells, a rarer type of stem cells, are able to differentiate into two or more cells within a specific cell lineage in a tissue. For example, hematopoietic stem cells (HSC) can differentiate into both myeloid and lymphoid lineages.^157^ HSC have been successfully employed to treat hematological malignancies and certain inherited metabolic diseases, but they cannot enhance solid organ regeneration.^16^ 5) Unipotent (or adult) stem cells can differentiate into only one specific cell type, such as satellite cells in the skeletal muscle.

The therapeutic effects of stem cell-based therapy can be generally attributed to three key mechanisms^158^: 1) Homing to the site of injury: certain stem cells, such as MSC, migrate to the site of injury due to chemotactic proteins binding to cell surface receptors (e.g., chemokine receptors), regardless of the location and method of injection.^159^ 2) Stem cell potency: The potency of a stem cell refers to its capacity to differentiate into different types of cells, which allows it to augment or replace injured tissues.^160^ 3) Secretion of bioactive factors: The secretion of bioactive factors mediates local and/or distant intercellular communication.^161^ Cells used for treating kidney disease include MSC,^9^ endothelial progenitor cells,^162^ primary renal cells,^163^ genetically modified cells,^164^ and iPSC.^165^ MSC are the predominant source of cells for CKD therapy.^9^ Early studies on MSC attributed their therapeutic potential to their ability to engraft, differentiate, and replace damaged tissues.^166^ However, it is well-established that long-term engraftment of MSC does not occur,^167^ and MSC do not remain in the tissue for more than 7–10 days after transplantation,^168^ which suggests that the main effects of MSC therapy are probably mediated through a paracrine mechanism.

Safety is another obstacle in the use of cell therapies in clinical applications. Risk factors of cell therapy can include^169^: 1) cell rejection or undesirable immune and inflammatory responses; 2) potential tumorigenicity or oncogenesis; 3) embolus formation; 4) adventitious agent or microorganism contamination; and 5) biodistribution or engraftment at undesired locations. A recent review outlined the safety concerns and adverse outcomes associated with the application of genetically modified cells in preclinical studies.^169^ Ninety-seven studies were included; however, only seven (7%) thoroughly reviewed the safety aspects of cell therapy. As it is difficult to assess all safety issues potentially associated with cell therapy, there may be health risks that have not yet been identified. Thus, stem cell-based therapies are facing a bottleneck in clinical transformation, and the ability to harness the paracrine effects of stem cell therapies without stem cells would provide a safer therapy for tissue repair and regeneration.

#### Exosome-based therapy

The use of cell-free exosome-based therapy provides several key advantages over stem cell-based therapy as it bypasses several safety concerns associated with stem cell transplantation.^158^ The first advantage is that the dosage and potency of exosomes can be evaluated in a manner similar to conventional pharmaceutical agents.^170^ Pharmacokinetic/pharmacodynamic testing of exosomes will allow personalized dosing according to patient needs. Second, exosomes can be effectively preserved for a relatively long period *in vitro*^171^ and can be potentially provided as off-the-shelf products since mass production is possible under controlled laboratory conditions.^158^ Finally, exosomes or parental cells can be modified or engineered to carry a specific cargo to obtain desired therapeutic effects.^172^

In recent years, various CKD preclinical models have been used to investigate the effects of exosomes on renal protection. However, to date, few published or ongoing clinical trials have tested the safety and efficacy of exosomes. One such ongoing study is examining EVs as biomarkers of chronic renal failure (https://www.clinicaltrials.gov; NCT04700631). A pilot study described the use of UMSC-derived EVs to ameliorate the progression of CKD; estimated GFR, serum creatinine, blood urea nitrogen levels, and urinary albumin/creatinine ratio were assessed after one year of administration.^173^ The results from this study suggest that UMSC-derived EVs can safely ameliorate immune system responses and successfully improve renal function in patients with grade III-IV CKD. However, long-term follow-up trials are required to confirm the safety and efficacy of exosome-based therapies.

### Limitation of exosomes

Although exosome-based therapy is a promising cell-free approach, several challenges still exist, thus, hindering its widespread application. 1) Standard techniques are required for high-yield and qualified exosome production, isolation and storage. Currently, these techniques vary among different studies,^174^ resulting in inconsistent reports of exosomal properties (e.g., morphology, size, concentration, purity, and potency). 2) Comprehensive pharmacological and preclinical studies are required to explore the best dosing time, means, duration, regimen, and potential detrimental roles of exosome therapy.^9^ For example, researches have demonstrated that some exosomal cargos (e.g., miR-320c) may also promote renal fibrosis.^175^ From this point, studies *in vitro*, animal experiment, and preclinical studies have to be quite solid before clinical trials to ensure that we have utilized exosomes in the renoprotective way. 3) The therapeutic effects of exosomes are limited by their rapid clearance and short half-life.^176^ Thus, further efforts should be made for extending effective duration. 4) The fate of exosomes after injection *in vivo* needs further investigation.^9^

## Improvement of exosome therapeutic activity

The improvement of exosome therapeutic activity contains two aspects: 1) increasing exosomal yield and 2) improving exosomal quality. Currently, preconditioning and engineering (eExos) are the two main strategies to improve the therapeutic activity of exosomes.^177^ Considering eExos have been mentioned above, in this section, we focus on the literature on preconditioning (Table 5).

**Table 5:** Preconditioning strategies for improving exosome therapeutic activity.

Category	Detailed cultural methods	Results
3D culture (Compared with 2D culture)	Scalable microcarrier-based 3D culture combined with TFF	Yield: 20–27 folds; Quality: more potent in siRNA transfer to neurons^179^
	Hollow fiber bioreactor-based 3D culture	Yield: 19.4 folds; Quality: more effective in attenuating pathological changes of renal tubules, inflammatory factors, T cell and macrophage infiltration in an AKI mouse model^180^
	Advanced 3D dynamic culture technique with exogenous TGF-β3 treatment	Yield: 33 folds; Quality: improved regeneration capacity^181^
Hypoxic culture (Compared with normoxia)	1% pO_2_	Yield: 2 folds; Quality: improved pro-angiogenic and renoprotective ability^183^
	<0.1% pO_2_	Quality: improved renoprotective effect against AKI^185^
	1% pO_2_	Quality: alleviated renal ischaemia-reperfusion injury though HIF-1α/Rab22 pathway^186^
Cytokine preconditioning	LPS (100 ng/ml)	Yield: increased the secretion of exosomes; Quality: contributed to macrophage polarization and inflammatory ablation^188^
	TNF-α (50 ng/ml) and IL-1α (50 ng/ml)	Quality: improved the angiogenic and regenerative potential of exosomes^187^
	IL-1β (10 ng/ml)	Quality: reduced inflammatory response^189^
Other chemical and physical preconditioning	Metformin (100 μg/ml)	Yield: facilitated exosome release Quality: increased exosomal protein content (involved in cell growth), and ameliorated intervertebral disc cells senescence^190^
	Acidic Ph	Yield: increased exosome production Quality: enhanced exosome stability, exosomal protein and RNA concentrations^191^
	Serum-free	Quality: enhanced pro-regenerative and pro-angiogenic bioactivities^192^

**Abbreviations**: 3D, three-dimensional; TFF, tangential flow filtration; siRNA, small interfering RNA; AKI, acute kidney injury; TGF-β3, transforming growth factor beta-3; TNF-α, tumor necrosis factor-α; IL-1α, interleukin-1α; LPS, lipopolysaccharide; IL-1β, interleukin-1β.

### 3D culture

The employment of 3D culture methods has positive effect on both exosomal yield and quality.^178^ Haraszti et al^179^ reported that 3D culture combined with tangential flow filtration increased the yield of exosomes with improved biologically activity. Similarly, Cao et al^180^ found that 3D-cultured MSC produced 15.5-fold exosomes than 2D culture, and enhances therapeutic efficacy of exosomes for AKI. Lim et al^181^ used an advanced 3D dynamic culture system combined with TGF-β3 to enhance exosome yield and efficacy.

### Hypoxic culture

Hypoxic incubation not only enhances exosome release,^182^ but also shows better therapeutic potential. It is demonstrated that hypoxia stimulates the production and secretion of exosomes in renal tubular cells.^183^^,^^184^ Additionally, Yu et al^185^ found that hypoxia-induced tubular exosomes were enriched with miR-20a-5p, and protected against AKI. Zhang et al^186^ reported that hypoxia-preconditioned renal tubular epithelial cell-derived exosomes alleviated renal ischaemia-reperfusion injury though HIF-1α/Rab22 pathway.

### Cytokine preconditioning

Cytokine^187^ and inflammatory stimulation^188^ were reported to improve exosome paracrine efficiency. Additionally, cytokines contribute to anti-inflammatory, pro-angiogenic, and pro-regenerative potential of exosomes. For instance, Kai Liu et al^189^ reported that exosomes isolated from interleukin-1β-treated MSC can reduce the inflammatory response of LPS-treated astrocytes. Gorgun et al^187^ found that the stimulation of TNF-α and IL-1α improved the angiogenic potential of exosomes.

### Other chemical and physical preconditioning

Different chemical and physical signals also affect the amount and content of exosomal secretion. For example, metformin facilitates the release of MSC-derived exosomes which ameliorate intervertebral disc cells senescence^190^; acidic pH enhances the stability and yield of exosomes, as well as exosomal protein and RNA concentrations^191^; exosomes derived from serum-free culture media result in enhanced wound healing and angiogenesis.^192^

Apart from preconditioning approaches mentioned above, it is easy to understand exosomes derived from early passaged cells or young donors were more efficacious than those derived from later-passaged cells or aged donors.^170^^,^^193^ Recent studies have demonstrated the tissue repair potential of exosomes generated from stem cells.^194^ Exosomes secreted by stem cells are the most efficacious in repairing their tissue of origin. Several surface ligands and adhesion molecules found on exosomes preferentially fuse or bind to receptors on similar cells, and the mechanism of exosomal uptake is source-dependent.^194^ For example, Schwann cell-derived exosomes but not fibroblast-derived exosomes can improve axonal regeneration.^195^ Although no studies have yet compared the use of renal cell-derived exosomes to exosomes derived from cells outside the renal system, one can postulate that exosomes from USC may provide a better therapeutic effect in the kidney than exosomes from other cell sources such as MSC.

## Future direction

Exosome therapy is a promising area of research for the treatment of renal fibrosis. Exosomes are small vesicles that can be derived from various cell types, and they contain proteins, RNAs, and other signaling molecules that can modulate various cellular processes, such as inflammation and tissue repair. Potential future developments for exosome therapy in the treatment of renal fibrosis include: 1) Identification of specific exosomal cargo: The ongoing research to identify specific molecules within exosomes are responsible for their therapeutic effects. By identifying these specific molecules, it may be possible to design more targeted exosome therapies that can selectively modulate specific cellular processes; 2) Optimization of exosome generation: Exosome production can be a time-consuming and costly process. Therefore, there is ongoing research to develop more efficient and scalable methods for exosome production, such as using microfluidic devices or bioreactors; 3**)** Combination therapies: Exosome therapy may be combined with other therapies, such as stem cell therapy, to enhance their therapeutic effects. For example, eExos may be used to deliver therapeutic molecules to the kidney, while the stem cells themselves may promote tissue repair; 4**)** Clinical trials: There are currently several ongoing clinical trials investigating the safety and efficacy of exosome therapy for the treatment of renal fibrosis. The results of these trials will provide valuable information on the optimal dose, timing, and route of administration of exosome therapy, as well as the potential side effects and long-term outcomes. Overall, exosome therapy has the potential to be a promising treatment for renal fibrosis. However, further research is needed to determine the optimal use of exosomes for the treatment of renal fibrosis and to evaluate their long-term safety and efficacy.

## Author contributions

Chuanqi Liu: Initial manuscript writing; Jian-Xing Ma and Baisong Lu: Modification of the manuscript; Tracy Criswell: Language polishing; Qingfeng Li and Yuanyuan Zhang: Conception formation; Management and coordination responsibility for the review execution.

## Funding

This project has been funded with the National Institute of Allergy and Infectious Diseases, National Institutes of Health, under Contract No. R21 AI152832, R03 AI165170 and R21 EY035833, the Wake Forest School of Medicine the TrEVR Center Translational Team Science Pilot Award 2024, and Eye Bank Association of America Research Grant Pilot Award 2024 (PI: Y. Z); Shanghai Clinical Research Center of Plastic and Reconstructive Surgery supported by Science and Technology Commission of Shanghai Municipality (Grant No. 22MC1940300); Shanghai Pujiang Program (2022PJD039).

## Conflict of interests

None.
